# Manipulation of photoassociation of ultracold Cs atoms with tunable scattering length by external magnetic fields

**DOI:** 10.1038/s41598-017-13534-6

**Published:** 2017-10-20

**Authors:** Guosheng Feng, Yuqing Li, Xiaofeng Wang, Jizhou Wu, Vladimir B. Sovkov, Jie Ma, Liantuan Xiao, Suotang Jia

**Affiliations:** 1 State Key Laboratory of Quantum Optics and Quantum Optics Devices, Institute of Laser spectroscopy, College of Physics and Electronics Engineering, Shanxi University, Taiyuan, 030006 P. R. China; 2 Collaborative Innovation Center of Extreme Optics, Shanxi University, Taiyuan, Shanxi 030006 P.R. China; 3 St. Petersburg State University, 7/9 Universitetskaya nab., St. Petersburg, 199034 Russia

## Abstract

We demonstrate that for ultracold, optically trapped Cs atoms the photoassociation (PA) can be manipulated by using external uniform magnetic fields due to the alteration of the scattering wavefunction in the region of the free–bound optical transition. We present PA–induced atom loss measurements with the same intensity for PA laser but different external magnetic fields, and analyze main contributions of the PA to the variation of the number of atoms in the trap. The PA rate exhibits a strong dependence on the changing uniform magnetic field. The experimental data are simulated within the model of a single–channel one–well rectangular potential, whose depth is adjusted so as to assure the predicted variation of the scattering length with the magnetic field. The computational and experimental results are in a reasonable agreement to each other. The same model is used to illustrate some general properties of the two–body quantum system in the near–threshold state.

## Introduction

Rapid progress has been witnessed in the formation and manipulation of ultracold molecules, and this is closely related to their wide applications over recent years^1^. At ultralow temperature, extremely precise control over molecular coherent dynamics can be exerted, and even the reactivities of such molecules can be steered at vanishing entropy^2^. Their rich internal structure makes them sensitive probes in precision measurements of fundamental physical constants up to a check of the hypothesis of the time variation of world constants^3–6^. Possibly strong dipolar interactions of heteronuclear dimers facilitate their application in quantum simulation of the strongly interacting regime and quantum computations^7,8^, and also enable proxy investigations of exotic condensed–matter phases^9^.

Laser–induced photoassociation (PA) and magnetic field–controlled Feshbach resonance are two typical ways in which ultracold atoms can be converted to the molecular bound states^10,11^. To effectively control over the atom-molecule conversion, an increasing number of theoretical and experimental researches provide new approaches to obtain an enhanced PA rate by increasing the density of atomic pairs in the short–range region^12–17^. A much–used method, investigated experimentally, is the Feshbach–optimized PA^18–20^, where PA had been also used to observe Feshbach resonances^21,22^. The near–Feshbach resonance wavefunction consists of strongly coupled bound and free hyperfine components of both single and triplet symmetry, resulting in greatly increased PA rates^23^. A similar mechanism is developed theoretically to obtain an efficiently direct stimulated Raman adiabatic passage of ultracold atoms in the continuum state to ground state molecules near a Feshbach resonance^24^. The efficient formation of ultracold molecules via the effective manipulation of PA enables the multi-purpose applications of ultracold molecules.

However, there are very few studies on the effect of uniform magnetic fields away from a Feshbach resonance position on the PA. These magnetic fields can be used to alter the atomic scattering length, which has a great effect on the PA rate^10,25^. In this paper, we present a feasable study on the efficient manipulation of the PA of the colliding pairs of ultracold Cs atoms by means of an external uniform magnetic field. Compared to the previous investigation^18^, our Cs atomic sample has a low temperature and is trapped in an optical dipole trap(ODT). Beside, we describe a theoretical model with the depth–adjustable square potential to match the magnetic field-induced variation of scattering length of atoms in their ground state, and calculate the Franck–Condon coupling strengths. Reasonable agreement between theory and experiment confirms that the shift of scattering phase of the colliding atom pairs induced by a uniform magnetic field gives rise to substantial change in the density of atomic pairs in the short–range region.

## Results

### PA of ultracold Cs atoms

**Figure 1:**
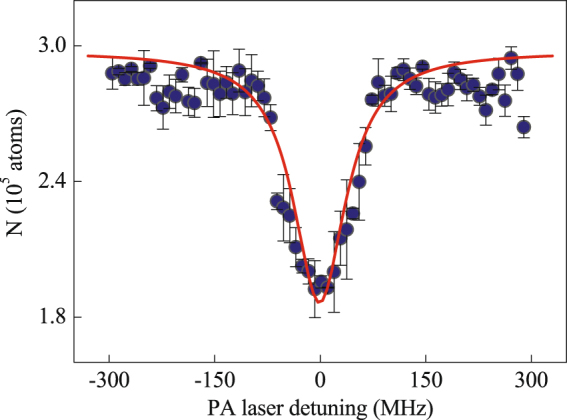
PA spectrum of ultracold Cs molecules in the *v* = 10, *J* = 0 rovibrational level in the outer potential of the long–range ${0}_{g}^{-}$ state with a double–well structure. The red line is a Lorentz fit of the observed data. The errors are mainly from the systematic uncertainty induced by the fluctuation of the number of optically trapped atoms in each experimental cycle and the uncertainty in the reading process of PA laser frequency.

The PA spectroscopy is measured by detecting the loss of Cs atoms optically trapped in a crossed ODT as a function of PA laser frequency. If the PA laser frequency is resonant with the energy difference between the scattering state of colliding atomic pairs and the bound excited state of diatomic molecule in a particular rovibrational level, the pure long-range state Cs_2_ molecule is produced in the outer well of the double–well potential of the ${0}_{g}^{-}$ (${6}^{2}{S}_{\mathrm{1/2}}+{6}^{2}{P}_{\mathrm{3/2}}$) state^26,27^. Figure 1 shows a typical PA spectrum of the rovibrational level $v=10$, $J=0$ of the electronically excited state of Cs_2_. The number of the Cs atoms remaining in the trap is detected by the standard absorption imaging method. We identify the frequency location of the maximum trap–loss as a position of the PA resonance, the resonant frequency is obtained as ~ 11672.098 cm^−1^ by applying the Lorentz fitting to the observed PA spectrum. The binding energy of this rovibrational level is directly inferred to be *E*_*bind*_ ~ −70.085 cm^−1^ when referring to the ${6}^{2}{S}_{\mathrm{1/2}}+{6}^{2}{P}_{\mathrm{3/2}}$ threshold.

We have investigated the influence of the magnetic field itself on the number of optically trapped atoms with a light field of an off-resonance frequency. For the Cs atoms in the hyperfine state $F=3$, ${m}_{F}=3$, the three-body loss rate is strongly dependent on the uniform magnetic field. However, the number of atoms remains almost unchanged with the changing magnetic field. A reasonable explanation is given by considering a large number of reduction of the atomic density in the plain evaporation process of 500 ms at a large scattering length of ~1250 ${a}_{0}$(Bohr radii) before the exposure of the atoms to the PA laser, where the three-body loss rate is usually proportional to both the fourth power of scattering length and the third power of the atomic density^28^. Thus, the dilute atomic sample makes it possible for the variation of $B$ to have almost no influence on the number of atoms in the ODT, and the PA is the main contribution to the loss of optically trapped Cs atoms.

### Dependence of PA rate on the magnetic field

**Figure 2:**
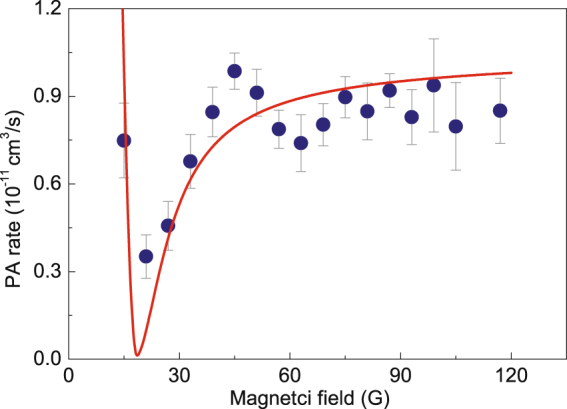
The on–resonance PA rate derived from the rate equation (1) for the atomic density, applied to the experimental atom loss, as a function of the magnetic field. The solid curve is obtained by the model calculation as described in the theory section.

The dependence of PA rate on the uniform magnetic field is investigated for a direct illustration to the manipulation of the PA using the uniform magnetic fields. The PA rate is known in terms of the on-resonance rate coefficient ${K}_{PA}$, which can be determined from the time evolution of the atomic density $n(r,t)$ in the PA process^29^. The relationship between the atom loss and the PA rate is described by the differential equation

1 $$\dot{n}(r,t)=-{K}_{PA}{n}^{2}(r,t),$$

implying that the atom loss mainly originates from the two-body PA process without an additional nontrivial three–body loss. In this case, we analytically solve for the time-dependent density distribution and extract ${K}_{PA}$ by spatially integrating the density and matching the observed and calculated atom loss after some time $\tau$. Compared to the previous experiments^19,29,30^, the exposure time $\tau$ of the PA laser on the atoms is longer than the trap oscillation period, thus any possible time dependence of ${K}_{PA}$ that deduced in this experiment is averaged over, and the atomic density is described without accounting for the details of the time evolution

2 $$n(\tau ,r)=\frac{n\mathrm{(0,}\,r)}{1+{K}_{PA}n\mathrm{(0,}\,r)\tau },$$

where $n\mathrm{(0,}\,r)$ is the initial density before switching the PA laser on, $\tau$ is the duration time of PA laser, and $n(\tau ,r)$ is the density after switching the PA laser off. The dependence of the PA rate ${K}_{PA}$ on the magnetic field is shown in Fig. 2, in which the variation of the PA rate with the magnetic field is corresponding but just opposite to the number of atoms in the crossed ODT, and this in turn demonstrates that the PA–induced two–body loss dominates the atom loss.

In Fig. 2, the theoretical curve shows reasonable agreement with the experimental result. The theoretical PA rate coefficient is estimated as the properly normalized squared overlap integral (Frank–Condon factor) of the model scattering wavefunction and the wavefunction of the upper bound state ${0}_{g}^{-}$ rovibrational level $v=10$, $J=0$. The atomic scattering wavefunction with the energy of ${k}_{B}\times 5$ *μ*K above the threshold is computed by using the analytical expressions of the square–well potential theory. The upper bound state wavefunction is computed with the standard Numerov algorithm using the potential function of the outer well of the ${0}_{g}^{-}$ state^31^.

## Theory model

**Figure 3:**
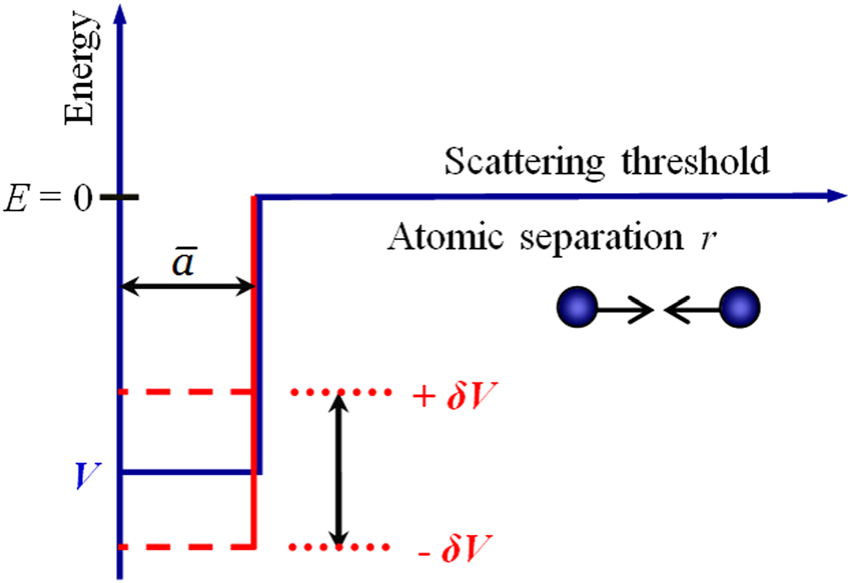
Illustration of the model depth–tunable square potential in a single channel system. The horizontal line in the outer region corresponds to the potential of *U* = 0; the dotted lines in the inner region indicate the depth of the potential tuned by the magnetic field so that to alter the scattering length and, consequently, the density of the atomic pairs in the short internuclear distance where PA occurs. *r* is the separation of two colliding atoms.

The theory of laser–assisted resonant cold collisions denotes that the strength of PA of ultracold atoms is determined by the coupling between the continuum wavefunction of the initially colliding atomic pairs and the wavefunction of the excited bound molecules under a PA laser field^25^. As has been already mentioned, the physically correct scattering wavefunction is presented by a mix of many channels formed by hyperfine components of the system. On the other hand, as is well known from the scattering theory^32^, the variation of the density of atomic pairs in short interatomic separations is strongly correlated with the asymptotic properties, such as the scattering length, which can be chosen as a governing parameter of the process, without much attention to the details of the short–range forces. This idea has been frequently exploited in the previous works^33,34^, where the simplified models of two coupled rectangular potentials were employed. The coupling of at least two channels looks to be important for a formation of the above–threshold Feshbach resonances, while the variation of atomic scattering wavefunction with the uniform magnetic field can be described within a simple model of a single–channel rectangular potential with an adjustable well depth as shown in Fig. 3.

Following refs^34,35^ we have chosen the radius of the model square–well potential to be the mean scattering length ${R}_{0}=\bar{a}=4\pi {\rm{\Gamma }}{\mathrm{(1/4)}}^{-2}{R}_{{\rm{vdW}}}$, where ${R}_{{\rm{vdW}}}=\frac{1}{2}{\mathrm{(2}{m}_{r}{C}_{6}/{\hslash }^{2})}^{\mathrm{1/4}}$ is the van der Waals radius and ${C}_{6}$ is the van der Waals coefficient at ${R}^{-6}$ in the realistic long–range potential. The mean scattering length of 95.7 *a*_0_ securely covers the range of an upper bound state wavefunction of excited Cs_2_ molecule in the long-range ${0}_{g}^{-}$ state.

The depth of the well for every magnetic field *B* is chosen so as to assure the predicted dependence^36,37^ of the scattering length *a* on *B*

3 $$a(B)=\mathrm{(1722}+1.52B\mathrm{)(1}-\frac{28.72}{B+11.74}),$$

where the units are the Bohr radius (a_0_) and Gauss (G). This equation must be valid for the ground state $F=3$, ${m}_{F}=3$ of the Cs atoms in the observed range of magnetic fields.

The global wavefunction of atoms in the entire region is constructed by the requirement for the wavefunction itself and its first derivative to be continuous at the switching point ${R}_{0}$; an additional condition is that the wavefunction turns to zero at $r=0$ in the inner region. Scattering state wavefunctions are conventionally normalized on the constant asymptotic amplitude, and bound state wavefunctions are normalized on the unit ${L}_{2}$ norm. The physically realizable bound states are those, for which $E < {V}_{\infty }=0$; this is only possible if

4 $$\tan ({k}^{+}{R}_{0})+{k}^{+}/{k}^{-}=0$$

where the superscripts “ + ” and “−” designate the states with the energy $E$ higher than and lower than the local value $V$ of the potential, ${k}^{\pm }=\sqrt{\mathrm{(2}m/{\hslash }^{2})|E-V|}$ is the local wavenumber. Sometimes in the scattering theory the virtual “antibound” states (not realizable physically) are also considered by $\tan ({k}^{+}{R}_{0})-{k}^{+}/{k}^{-}\,=\,0$.

The scattering length $a$ can be obtained via the coefficients of the threshold ($E={V}_{\infty }=0$) solution. Hence, the wavenumber in the inner region of the asymptotically threshold state obeys the equation

5 $$\tan ({k}^{+}{R}_{0})/{k}^{+}={R}_{0}-a.$$

After solving the latter equation for ${k}^{+}$, the well depth $V=({\hslash }^{2}\mathrm{/2}m){k}^{+2}$, providing the desired value of the scattering length $a$, can be found.

**Figure 4:**
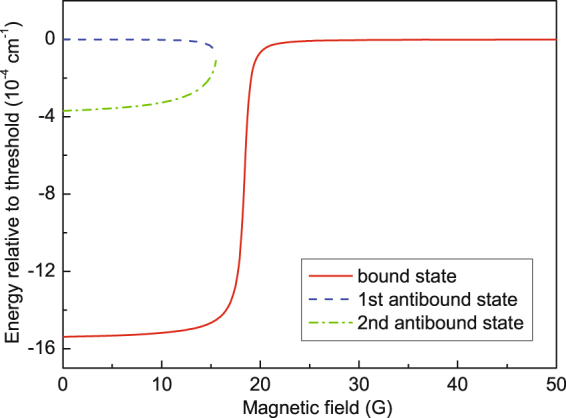
Energies of the bound and virtual antibound states in the rectangular potential relative to the threshold as functions of magnetic field.

However, due to a periodicity of the tan function, the solution of this equation is not unique. The physical reason for this is that the desired variation of the scattering length is governed by the properties of a near–threshold bound or antibound level, independently of how many states lay below this near–threshold level. In most of our calculations we have chosen the shallowest well containing only one bound state level. We do the model calculations based on the above theory and Eq. 3 for the scattering length. The most principal results of the modeling are shown in the theoretical variation of PA rate with the uniform magnetic field as shown in Fig. 2. The depth of the potential well decreases with the increasing magnetic field. As indicated in Fig. 4, a near-threshold bound state produces a big positive scattering length; a near–threshold antibound state produces a big negative scattering length. The big absolute value of the scattering length indicates the enhancement of the spatial density (wavefunction amplitude) of near–threshold states.

## Discussion

**Figure 5:**
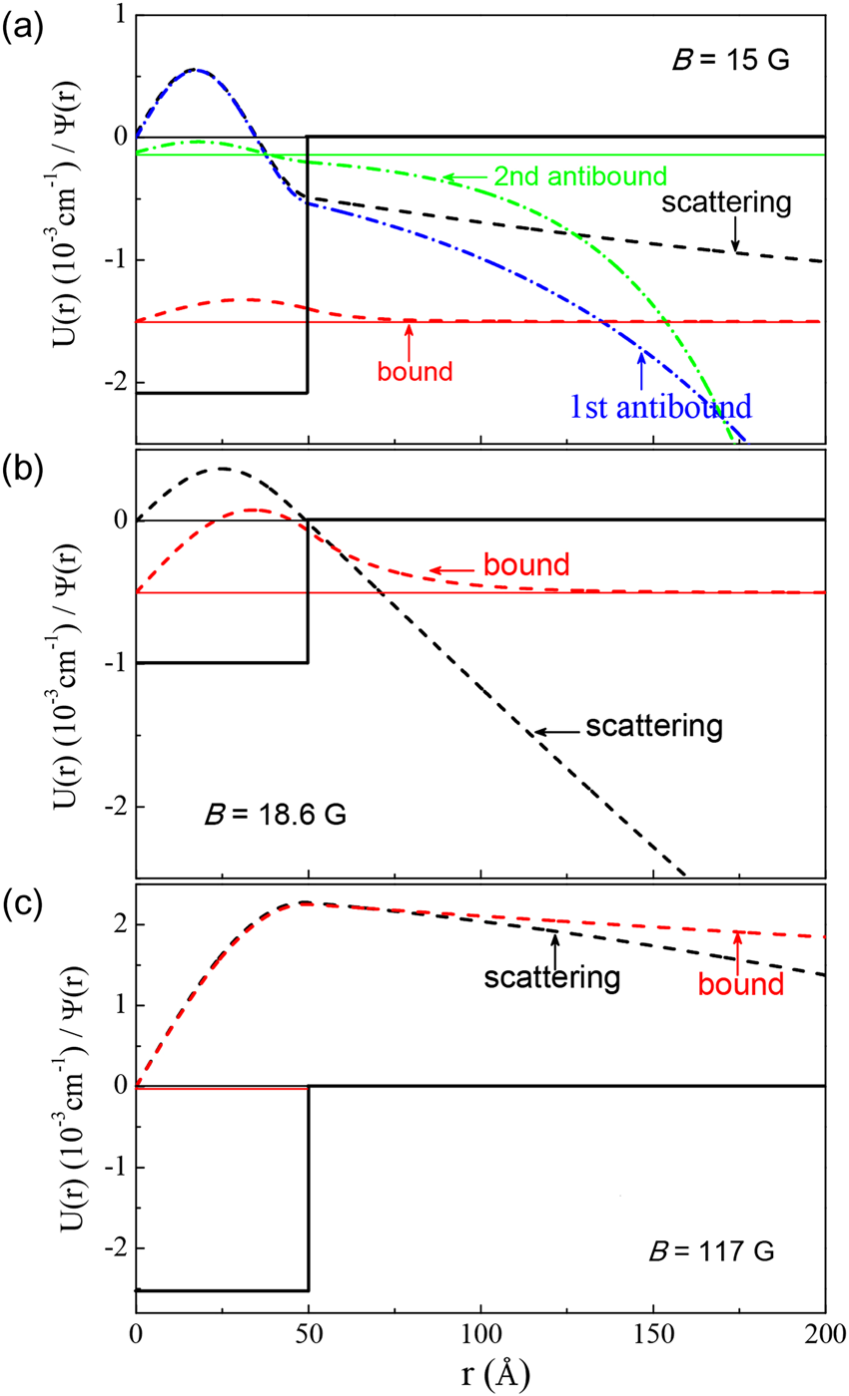
The potential well and the wavefunctions at the magnetic fields of *B* = 15 G (**a**), 18.6 G (**b**) and 117 G (**c**).

To clarify the underlying physical mechanism, we discuss some intermediate results. Figure 4 shows the energies of the bound and antibound states relative to the threshold as functions of the magnetic field within the most representative range. We see that, besides the only one bound state, there exist two antibound states, which coalesce and vanish at a switching point. To the left of this point the antibound states lay higher than the bound one, so the upper of them affects the near–threshold scattering behavior in a first place. As soon as the bound state approaches the threshold to the right of this point, it begins to determine the scattering behavior. These properties are illustrated in Fig. 5, showing the square–well potential function and the wavefunctions (arbitrary normalized) of the scattering, bound, and antibound states at various values of magnetic field. The variation of the energies of the bound and antibound states in Fig. 4 also is consistent to the appearance of PA rate in Fig. 2.

In Fig. 5(a) with the magnetic field of $B=15$ G, one of the antibound levels lays close to the threshold and, correspondingly, its wavefunction is very similar to the scattering state wavefunction, at least in the inner region. This causes a relative enhancement of the PA rate displayed in the left part of Fig. 2. Figure 5(b) shows the corresponding results for *B* = 18.6 G, at which a very small PA rate coefficient is observed in Fig. 3. The reason is that there neither bound nor antibound near–threshold state exists: the antibound states have already gone, while the bound state is still rather far from the threshold. The resemblance between the bound and scattering state wavefunctions is not as good as in the other cases. When the magnetic field is tuned to the value of *B* = 117 G, the bound state level is very close to the threshold, its wavefunction is very similar to the scattering one, as shown in Fig. 5(c), and the PA rate increases again.

The widely used approximate expression for the scattering length via the binding energy of a near–threshold bound/antibound state is given as

6 $$|a|\approx \mathrm{1/}{k}^{-}=\sqrt{{\hslash }^{2}\mathrm{/(2}m|E-V|)}.$$

**Figure 6:**
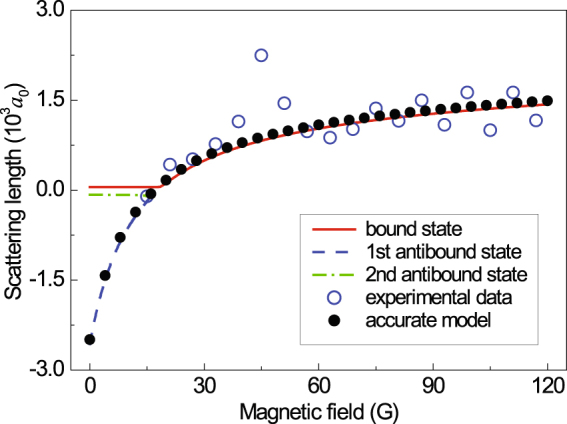
The scattering lengths as functions of the magnetic field: accurate model, estimated from the binding energies of the bound and virtual antibound states, and the ones estimated from the experimental data within a framework of the model of the rectangular potential.

We have compared this estimate to the accurate model scattering length in Fig. 6. The experimental values, also shown here, have been computed by fitting our model to the experimental PA rates. We see that Eq. 6 produces reasonable estimates for the scattering length if only there indeed a near–threshold bound or antibound state presents; however, it breaks in the region of no such state. There is a small peak for the experimental scattering length and this local deviation may be attributed to the perturbation from the narrow $d$-wave Feshbach resonance at the magnetic field of $B$ = 47.9 G. Remaining discrepancies, noticeable at high magnetic fields, can be caused by a shallowness of the well, as the inequality $|E|\ll |V|$ is definitely a condition for Eq. 6 applicability. Although our single-channel square-well potential model doesn’t include the bound state of Feshbach molecule, it can be used to give the effective wavefunction of two colliding atoms in the short separation and then to calculate the overlap between the atomic scattering states and excited bound molecular states.

## Methods

### Preparation of ultracold Cs atom sample

The experimental study on the manipulation PA of optically trapped ultracold Cs atoms using a uniform magnetic field is based on the trap–loss spectroscopy of the ultracold atomic sample at the changing magnetic field. To obtain an ultracold atomic sample, the three–dimensional (3D) degenerated Raman sideband cooling (DRSC) is employed to cool and polarize the Cs atoms in the desired state $F=3$, ${m}_{F}=3$ ^38^. After 10 ms of 3D DRSC, we obtain $1.5\times {10}^{7}$ atoms with the temperature of ~1.7 *μ*K^39^. Subsequently, the cooled atoms are loaded into a large–volume crossed ODT operating at 1070 nm. The crossed ODT creates a confined potential of $\approx -{k}_{B}\times 45$ *μ*K for Cs atoms. Due to a large anti–trapping potential induced by the gravity, the magnetic field gradient of 31.13 G/cm is produced using a pair of coils in the anti–Helmholtz configuration for the magnetically levitated loading^40^. Meanwhile, another pair of coils in the Helmholtz configuration provide the magnetic field of 75 G to cancel the resulting anti–trapping potential in the horizontal direction from the application of the magnetic field gradient.

### Experimental procedure of PA at different magnetic fields

Once the atoms are prepared in the crossed ODT, PA is performed by illuminating the atoms with a certain detuning to the Cs ${D}_{2}$ transition ${6}^{2}{S}_{\mathrm{1/2}}\to {6}^{2}{P}_{\mathrm{3/2}}$ near the wavenumber of 11672 cm^−1^. The corresponding PA laser is obtained from a widely tunable continuous–wave (cw) Ti:sapphire laser, which yields an output power of 1 W with a narrow linewidth of ~75 kHz. The long–time frequency drift of the PA laser is less than 500 kHz by locking it to its self–reference cavity. After optical alignment and fiber coupling, a beam with the power of 108 mW is focused to a waist of ${\omega }_{0}$ = 150 *μ*m, resulting in the intensity of $I=141.5$ W/cm^2^. After 500 ms of the plain evaporation dominated by a three-body loss at the *B* of 75 G, the PA laser is switched on and the atomic cloud is illuminated for 100 ms.

For investigating the effect of external uniform magnetic fields on the PA, we measure the PA of Cs atoms at different magnetic fields. Prior to switching on the PA laser focused on the Cs atoms, the magnetic field is ramped up or down over 30 ms to a magnetic field *B*, and afterwards the PA of the Cs atoms is performed for 100 ms at this *B* field. The dependence of the PA rate on the magnetic field is determined by recording trap loss spectra at different magnetic fields. However, the variation of the magnetic field induces the shift of the resonant frequency between the scattering atomic and excited bound molecular states due to the Zeeman effect of the hyperfine state $F=3$, ${m}_{F}=3$ of Cs atoms. Thus, the PA laser frequency is tuned with a few megahertz to compensate the shift of the resonant frequency, which is twice as much as the Zeeman shift.

## Conclusions

To summarize, we show that it is possible to use an external uniform magnetic field to manipulate the PA of ultracold atoms optically trapped in a crossed dipole trap, that, subsequently, makes it possible for enhancing the PA used to form ultracold molecules. The experimental results are reproduced with a reasonable quality by using the model of a single–channel square–well potential with the depth adapted to the magnetic field via the scattering length as a governing parameter. We present the clear physical mechanism for efficiently manipulating PA, the spatial density of near-threshold states in the short interatomic separation region can be manipulated by altering the scattering length that is related to the near-threshold bound and anti-bound states. Our result demonstrates that the one-channel square potential not only provides a tool for a reasonable modeling of the PA rate observed in our experiment, but can also serve as a very clear and well understandable illustration of the general near–threshold physics in a two–body quantum system.

## References

[1] Krems, R. V., Stwalley, W. C. & Friedrich, B. Cold Molecules: Theory, Experiment, Applications. CRC Press (2009).

[2] Krems RV (2005). Molecules near absolute zero and external field control of atomic and molecular dynamic. Int. Rev. Phys. Chem..

[3] Zelevinsky T, Kotochigova S, Ye J (2008). Precision test of mass–ratio variations with lattice confined ultracold molecules. Phys. Rev. Lett..

[4] DeMille D (2008). Enhanced sensitivity to variation of *me*/*m*_*p*_ in molecular spectra. Phys. Rev. Lett..

[5] Beloy K, Borschevsky A, Flambaum VV, Schwerdtfeger P (2011). Effect of *α* variation on a prospective experiment to detect variation of *m*_*e*_/*m*_*p*_ in diatomic molecules. Phys. Rev. A.

[6] Sainis S (2012). Detailed spectroscopy of the Cs_2_ a^3^Σ^+^_*u*_ state and implications for measurements sensitive to variation of the electron–proton mass ratio. Phys. Rev. A.

[7] Micheli A, Brennen GK, Zoller P (2006). A toolbox for lattice–spin models with polar molecules. Nat. Phys..

[8] DeMille D (2002). Quantum computation with trapped polar molecules. Phys. Rev. Lett..

[9] Baranov MA (2008). Theoretical progress in many–body physics with ultracold dipolar gases. Physics Reports.

[10] Jones KM, Tiesinga E, Lett PD, Julienne PS (2006). Ultracold photoassociation spectroscopy: Longrange molecules and atomic scattering. Rev. Mod. Phys..

[11] Chin C, Grimm R, Julienne PS, Tiesinga E (2010). Feshbach resonances in ultracold gases. Rev. Mod. Phys..

[12] Kallush S, Kosloff R (2007). Momentum control in photoassociation of ultracold atoms. Phys. Rev. A.

[13] Pellegrini P, Gacesa M, Côté R (2008). Giant formation rates of ultracold molecules via Feshbach-optimized photoassociation. Phys. Rev. Lett..

[14] Mackie M, Fenty M, Savage D, Kesselman J (2008). Cross–molecular coupling in combined photoassociation and Feshbach resonances. Phys. Rev. Lett..

[15] Zhang W, Wang GR, Cong SL (2011). Efficient photoassociation with a train of asymmetric laser pulses. Phys. Rev. A.

[16] González-Férez R, Koch CP (2012). Enhancing photoassociation rates by nonresonant–light control of shape resonances. Phys. Rev. A.

[17] Hu XJ, Xie T, Huang Y, Cong SL (2014). Feshbachoptimized photoassociation controlled by electric and magnetic fields. Phys. Rev. A.

[18] Tolra BL (2003). Controlling the formation of cold molecules via a Feshbach resonance. Europhys. Lett..

[19] Junker M (2008). Photoassociation of a Bose-Einstein condensate near a Feshbach resonance. Phys. Rev. Lett..

[20] Krzyzewski SP, Akin TG, Dizikes J, Morrison MA, Abraham ERI (2015). Observation of deeply bound ^85^*Rb*_2_ vibrational levels using feshbach optimized photoassociation. Phys. Rev. A.

[21] Courteille P, Freeland RS, Heinzen DJ (1998). Observation of a Feshbach resonance in cold atom scattering. Rhys. rev. Lett..

[22] Chin C, Kerman AJ, Vuletić V, Chu S (2003). Sensitive detection of cold cesium molecules formed on Feshbach resonances. Phys. Rev. Lett..

[23] Gacesa, M., Ghosal, S., Byrd, J. N. & Côté, R. Feshbach–optimized photoassociation of ultracold ^6^*Li*^87^*Rb* molecules with short pulses. *Phys. Rev. A* **88**, 063418 (2013).

[24] Kuznetsova, E., Gacesa, M., Pellegrini, P., Yelin, S. F. & Côté, R. Efficient formation of ground–state ultracold molecules via STIRAP from the continuum at a feshbach resonance. *New J. Phys.* **11**, 055028 (2009).

[25] Bohn JL (1999). & Julienne, P. S. Semianalytic theory of laser–assisted resonant cold collisions. Phys. Rev. A.

[26] Fioretti A (1999). Photoassociative spectroscopy of the long-range state. Eur. Phys. J. D.

[27] Ma J (2014). New observation and combined analysis of the Cs_2_ 0^−^_*g*_, 0^+^_*u*_, and 1_g_ states at the asymptotes 6S_1/2_ + 6P_1/2_ and 6S_1/2_ + 6P_3/2_. J. Chem. Phys..

[28] Weber T, Herbig J, Mark M, Nägerl H-C, Grimm R (2003). Three-body recombination at large scattering lengths in an ultracold atomic gas. Phys. Rev. Lett..

[29] McKenzie C (2002). Photoassociation of sodium in a Bose-Einstein condensate. Phys. Rev. Lett..

[30] Prodan ID, Pichler M, Junker M, Hulet RG, Bohn JL (2003). Intensity dependence of photoassociation in a quantum degenerate atomic gas. Phys. Rev. Lett..

[31] Bouloufa N, Crubellier A, Dulieu O (2007). Reexamination of the 0^−^_*g*_ pure long-ange state of Cs_2_: Prediction of missing levels in the photoassociation spectrum. Phys. Rev. A.

[32] Friedrich, H. Scattering Theory. Springer, The Netherlands. (2003).

[33] Kokkelmans SJJMF, Milstein JN, Chiofalo ML, Walser R, Holland MJ (2002). Resonance superfluidity: Renormalization of resonance scattering theory. Phys. Rev. A.

[34] Lange AD (2009). Determination of atomic scattering lengths from measurements of molecular binding energies near Feshbach resonances. Phys. Rev. A.

[35] Gribakin GF, Flambaum VV (1993). Calculation of the scattering length in atomic collisions using the semiclassical approximation. Phys. Rev. A.

[36] Chin C (2004). Precision Feshbach spectroscopy of ultracold Cs_2_. Phys. Rev. A.

[37] Kraemer T (2006). Evidence for Efimov quantum states in an ultracold gas of cesium atoms. Nature.

[38] Kerman AJ, Vuletić V, Chin C, Chu S (2000). Beyond optical molasses: 3D Raman sideband cooling of atomic cesium to high phase-space density. Phys. Rev. Lett..

[39] Li Y (2015). Enhanced Raman sideband cooling of caesium atoms in a vapour-loaded magneto-optical trap. Laser Phys. Lett..

[40] Li YQ (2015). Magnetic levitation for effective loading of cold cesium atoms in a crossed dipole trap. Phys. Rev. A.

